# Polyethylene-Carbon Composite (Velostat^®^) Based Tactile Sensor

**DOI:** 10.3390/polym12122905

**Published:** 2020-12-03

**Authors:** Andrius Dzedzickis, Ernestas Sutinys, Vytautas Bucinskas, Urte Samukaite-Bubniene, Baltramiejus Jakstys, Arunas Ramanavicius, Inga Morkvenaite-Vilkonciene

**Affiliations:** 1 Department of Mechatronics, Robotics, and Digital Manufacturing, Vilnius Gediminas Technical University, J. Basanaviciaus Str. 28, LT-03224 Vilnius, Lithuania; andrius.dzedzickis@vgtu.lt (A.D.); ernestas.sutinys@vgtu.lt (E.S.); vytautas.bucinskas@vgtu.lt (V.B.); urte.samukaite-bubniene@vgtu.lt (U.S.-B.); baltramiejus.jakstys@vdu.lt (B.J.); 2 Department of Physical Chemistry, Faculty of Chemistry, Vilnius University, Naugarduko Str. 24, LT-03225 Vilnius, Lithuania; 3 Laboratory of Nanotechnology, Center for Physical Sciences and Technology, Sauletekio Av. 3, LT-10257 Vilnius, Lithuania; 4 Laboratory of Electrochemical Energy Conversion, Center for Physical Sciences and Technology, Sauletekio av. 3, LT-10257 Vilnius, Lithuania

**Keywords:** tactile sensors, pressure/force sensors, conducting polymers, polymers, Velostat^®^

## Abstract

The progress observed in ‘soft robotics’ brought some promising research in flexible tactile, pressure and force sensors, which can be based on polymeric composite materials. Therefore, in this paper, we intend to evaluate the characteristics of a force-sensitive material—polyethylene-carbon composite (Velostat^®^) by implementing this material into the design of the flexible tactile sensor. We have explored several possibilities to measure the electrical signal and assessed the mechanical and time-dependent properties of this tactile sensor. The response of the sensor was evaluated by performing tests in static, long-term load and cyclic modes. Experimental results of loading cycle measurements revealed the hysteresis and nonlinear properties of the sensor. The transverse resolution of the sensor was defined by measuring the response of the sensor at different distances from the loaded point. Obtained dependencies of the sensor’s sensitivity, hysteresis, response time, transversal resolution and deformation on applied compressive force promise a practical possibility to use the polyethylene-carbon composite as a sensitive material for sensors with a single electrode pair or its matrix. The results received from experimental research have defined the area of the possible implementation of the sensor based on a composite material—Velostat^®^.

## 1. Introduction

Polymer-based materials and composites are very promising for the development of various tactile sensors [1,2,3]. Such sensors require very stable and reliable polymeric materials and efficient and accurate systems for the transduction of analytical signal [4]. In sensor design, conducting and semiconducting polymers are successfully applied due to their versatile properties and stimuli-dependent electrical conductivity and other electrical or electrochemical characteristics [5,6,7,8].

One of the most stable, reliable and, therefore, probably the most promising polymeric composite material for the design of tactile sensors is Velostat^®^, also known as Linqstat [9]. The main advantages of this material are flexible range of dimensions, mechanical and chemical stability and relatively low price [10]. Velostat^®^ is a composite polymer material consisting of carbon-impregnated polyethylene. Entrapped carbon powder turns initially dielectric polyethylene into electrically conducting composite material which belongs to the group of piezoresistive materials. It can be used for various purposes, especially in the design of flexible sensors, which are highly demanded for mechatronics and biomedical application [10,11,12]. The typical application field of flexible piezoresistive sensors covers the evaluation of pressure or applied normal force in non-stiff objects, such as normal stress in various materials [13], normal soil stresses [14], normal stresses in other objects with high lateral deformation [15,16]. Flexible piezoresistive sensors can be applied in robotics [17,18,19], wearable sensors [20] and human-machine interaction devices [21]. However, the implementation of a piezoresistive sensors in mechatronic and robotic systems is still limited by the lack of reliable materials, which have stable characteristics [22]. Moreover, all these applications require the elaboration of suitable scientific and engineering approaches for the creation of all classes of here-mentioned tactile sensors. Some applications are already known, but many applications in the field, especially in mechatronics and robotics, are not well covered yet.

The main disadvantage of the most piezoresistive materials is that their electrical properties changes with material ageing. Piezoresistive materials deform in time and do not return to their initial state. Moreover, piezoresistive sensors usually suffer from nonlinearity and exhibit significant hysteresis [23]. The structure of a material is non-homogenous, and this feature has an impact on the repeatability of measurement data. All these limiting factors result in poor accuracy, repeatability, and ability to be easily replaced, which makes these sensors unsuitable for precise, repeatable and reliable pressure or force measurements.

Moreover, due to the multidirectional (normal and transversal) conductivity of the Velostat^®^, the application of this material for sensor arrays becomes complicated. The pressure applied to one array point affects the resistance not only at a single point but also on many adjacent points. This phenomenon can be explained by the fact that electric current flows through the path with the least resistance. This problem usually is solved by performing precise sensor array calibration under various conditions or implementing smart algorithms [24] for data processing and analysis. Overcoming these problems will increase the application area of Velostat^®^, especially towards the application of this composite polymer in high-performance sensors. Therefore, detailed research of its characteristics is required, and algorithms to compensate their uncertainties needs to be elaborated and implemented.

The most significant advantages of resistive sensors comparing them to the sensors of other types are low manufacturing cost, simplicity of interfacing circuits and data acquisition process. The possibility to adjust sensor measurement range for required load within varying its geometric size and larger surface area allows measuring higher distributed load. Implementations of Velostat^®^ material as a sensitive layer of the sensors in applicable devices have been reviewed in many studies. Bibliographical sources of such researches are provided in the Table 1. Recently, Velostat^®^-based structures were implemented for various applications, but the evaluation of Velostat^®^ electrical and mechanical characteristics is still insufficient.

The sensitivity of the Velostat^®^ to the applied pressure is mainly defined by two physical phenomena: quantum tunneling and percolation [23]. Quantum tunneling uses the tunnel effect that affects the conductivity of a composite material when the distance between conductive particles inside polymeric materials varies due to the applied pressure, which deforms the material. Conductive particles can be electrically connected prior to geometric connection. This means that although the particles have no physical interaction within geometry, they do interact “electrically” through the interparticle tunneling effect. Therefore, percolation is a function of geometrical features. Percolation is linked to a change of conductivity between the isolating and conductive state of a material caused by the change of applied pressure [25]. The conductance of the material varies due to the change of the contact area (caused by the material deformation) of conductive particles; in this way, direct conductive paths are created inside the composite material [12]. Velostat^®^ changes electrical resistance due to both above mentioned effects, when mechanical bending, tension, or pressure are applied [23].

Percolation describes the connections of disordered objects in the matrix structure and the influence of these connections on the properties of the system at the macro scale. The percolation threshold ascribes the minimum filler content in the polymer matrix after which there is no significant change in the electrical properties of the system. In the case of polymeric composites with conductive fillers, the system can be considered as a randomly arranged conductive element in the dielectric material. In this case, the electric current can flow only between connected groups of connected elements. The threshold of electrical percolation in polymer composites depends mainly on the characteristics of the conductive filler, such as size, density, length-to-diameter ratio, electrical properties, location and orientation in the polymer matrix and other aspects.

The polyethylene-carbon matrix percolation has been investigated and reviewed in various [26,27,28,29,30] papers. In a detailed experimental study, it was demonstrated that the percolation threshold of polypropylene based nanocomposites with exfoliated graphite nanoplatelets (GnP), carbon black (CB), vapor grown carbon fillers (VGCF) and polyacrylonitrile (PAN) carbon fibers as fillers depends on a variety of factors, including filler characteristics (percolation threshold: 2 vol% for CB, 5%–8% for VGCF, 8%–9% for GnP, 8%–10% for PAN based carbon fibers filler composition) and fabrication conditions. Crystallization characteristics of the polymer during the molding process can be tuned within the cooling rate [26].

Carbon particles localized at the interface of polymers normally introduce a low percolation threshold—0.2–0.3 vol% [31]. In the case of aggregated carbon black or carbon fibers in the high density polyethylene matrix [24] the conductive network forms and when the short-cut carbon fibers volume fraction is 20%, the value of resistivity of the system is about 8.6 Ω·cm. The percolation threshold value is close to 7.5 vol% [27]. The role of multiaxial orientation in low-density polyethylene filled with carbon fiber tubes is highlighted in [29] paper. The percolation threshold decreases from 35 wt% in uniaxial tubes to 15 wt% in rotation-extruded ones. The percolation threshold of conventional polypropylene-carbon black composites was calculated and is about 2.75 vol%. In another study, polypropylene-carbon conductive composites with very low percolation threshold (0.37 vol%) was obtained by constructing a particular continuous segregated structure [32].

In the case of the loaded weight, the higher the load, the higher the conductivity, and the lower the resistance as the path of electrons shortens. The lower disordering degree has a higher free path distance of electrons—the carbon layer became denser, and the conductive network for electron transfer is well built. The polyethylene matrix maintained its structure and mechanical properties. The higher load (more than 3 N) could cause damage to Velostat® and lead to the loss of conductivity or shortening of the measurement circuit.

The applied load up to 3 N re-deforms the Velostat^®^ material, thus shortening the path of the electrons, as a result of which the relative resistance decreases. Results of sensor sensitivity evaluation (Figure 5) reflect the nonlinear behavior of the sensors, which corresponds to its mechanical properties. It is seen that in a comparison between fresh and used polyethylene-carbon material, the resistance of fresh material is higher. It can be stated that this change is caused by the previously described effect: applied load causes a decrease of the material thickness and the squeezing of carbon grains deeper into the material. This effect decreases the distance between the conductive filler particles and increases the conductivity of polyethylene-carbon material.

Properly dispersed filler along the polymeric matrix improves chemical and mechanical properties. The mechanical properties of carbon derivatives depend on (i) aspect ratio (l/d), (ii) concentration, (iii) dispersion and orientation, geometry, (iv) conjunction between the filler and the polymeric matrix.

However, none of the models that can predict and describe the percolation threshold as a function of the influencing factors can be used because of the limitations—(i) it is hard to investigate all the factors that affect the conductivity and (ii) empirical parameters that cannot be measured experimentally need to be explored [33].

The sensor design is an important feature which allows the use of the same piezoresistive material for different applications by varying its volume, surface area, shape, and distribution of electrodes. The measurement range and sensitivity of such sensors usually depends on the mechanical characteristics of a used piezoresistive material, for example, if it is necessary to measure higher loads, volume and surface area of sensitive material should be increased [34]. In this research, a sensor with 25 mm^2^ surface area was selected. Such an area size fits the load up to 3 N measurements. The higher load could cause damage to Velostat^®^ and lead to the loss of conductivity or shortening the measurement circuit. When selecting the size of the sensor surface, it is also necessary to take into account the expected sensor resistance under the maximum load. The lower the resistance, the lower measurement accuracy could be expected. If resistance turns lower than a few hundred ohms than the resistance of the connection circuit will have a significant effect on the accuracy of measurement results. Supplementing the selection, a sensor with a surface area of 25 mm^2^ is relatively compact and suits various applications: it could be installed into robot small grippers for clamping force sensing; it could be used for object detection, and object recognition based on weight evaluation; it could be used as one element of a sensor array in various pressure mapping applications. The stability of the measurement results depends mainly on the fluctuations of power supply and drift of the reference resistors resistance value as well as the electrical properties of a sensitive material. Issues related to the power supply can be solved quite easily by the selection of a suitable power supply unit with corresponding power requirements and stabilized output voltage. The drift of the reference resistors mainly depends on the resistor’s thermal sensitivity; therefore, when designing a measurement circuit, it is essential to evaluate the value of the current in the circuit and minimize it as much as possible to prevent reference resistors from self-heating. The accuracy of the measurements mainly depends on the precise evaluation of the reference resistance and supply voltage values. Those values have a direct impact on the evaluation of electrical resistivity of the polymeric composite.

Moreover, it is essential to note that it relays only on the determination of resistance. In the most practical cases, it is necessary to recalculate the obtained resistance into pressure or acting force. Such a conversion brings additional errors, which mainly depend on the accuracy of the resistance/pressure approximation model and non-homogeneity of the applied polymeric composite material.

The carried-out in-depth literature analysis revealed the lack of research on mechanical properties of the Velostat® layer in the sensor matrix. The mechanical properties of Velostat® have an influence on the electrical properties of the sensor. Our paper covers the analysis of the behavior of this material under the long term and repeating load, therefore we can apply the model of elasto-plastic material for Velostat®. The correctly selected material model can ensure suitable load and measurement modes and avoid undesirable loading cases and errors in measurements. The novelty of our research is the instant measurement of the electric and mechanical properties of the Velostat® with various load cases. The influence of the material surface roughness pattern cannot be neglected too—fresh material has much higher peaks than used material, therefore the strain of the material differs during the sensor’s lifetime. Fortunately, surface topology morphs to steady-state after a few loadings; therefore, stable output for the sensor is possible through its lifetime.

Another big issue in creating tactile sensors is using matrixes of sensors formed on the same piece of the Velostat^®^ sheet. This technology opens a broad perspective to detect the touch or load distribution in the sensor matrix area. We performed the experimental evaluation of possible sensor resolution or matrix element distance, by loading neighboring areas of material. Such research is new, no similar experiment has been done, and there are currently no reports available.

Therefore, the aim of this study is to evaluate the characteristics of the polymeric composite material Velostat^®^ and to define the most methods for practical application of this composite material in tactile sensors.

## 2. Experimental

Progress in the application of tactile sensors depends on the selection of composite materials and their characteristics, implementing the usage of the composite for various purposes. Two types of characteristics (mechanical and electrical) were analyzed in this research.

Mechanical characteristics define a profile of a sensor for repeatability, loss of sensitivity over a long time period and its restoring pattern. To investigate these parameters, a series of experiments was performed by measuring normal deformation of a material dependent on incremental loading. Long term loading and relaxation processes reveal material lifetime-related issues. Mechanical settling of a material is important for steady sensor operation, and it was evaluated by measurement of stiffness and internal friction of the Velostat^®^.

Electrical properties of the sensor material were tested by evaluating the non-linearity of resistance and quantitative evaluation of hysteresis, together with settling parameters of the material after a few trials. The confidence interval of measurements was defined by electrical resistance fluctuations during measurements in a cyclical manner. Another critical issue tested in this experimental research was the resolution of the sensor evaluated by the influence of a locally applied load on a distance between parallel contacts. This effect is caused within the independently created force and the level of their internal interference measurements.

The experimental research of the developed resistance sensor with piezoresistive material was performed in four stages: (i) the evaluation of the developed sensor displacement dependent on the compressive force; (ii) the determination of the sensor voltage dependent on the compressive force; (iii) the detection of sensor response time dependent on voltage; (iv) the measurement of voltage dependent on the distances between the contacts of the developed sensor.

### 2.1. Equipment

**Figure 1:**
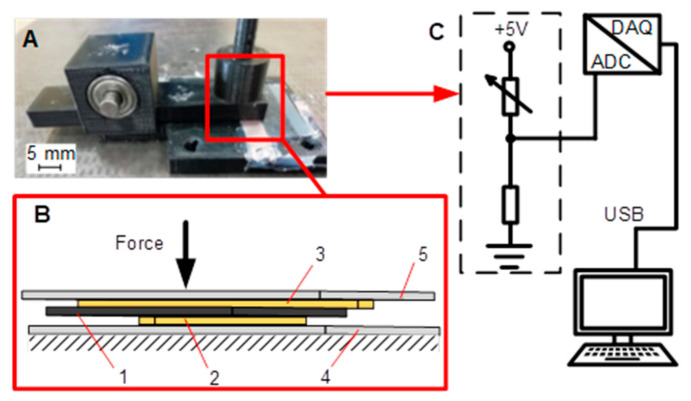
Design of the test rig used for experiments. (**A**) Common view; (**B**) Design of the sensor; (**C**) Electrical scheme. 1—pressure-sensitive conductive material Velostat^®^, 2—fixed bottom electrode, 3—upper electrode, 4—fixed test rig plate, 5—movable test rig plate.

The design of the test rig used for experiments is provided in Figure 1. The sheet of the pressure-sensitive conductive material Velostat^®^ (1) was placed between two copper electrodes 50 × 5 × 0.2 mm (2 and 3), the bottom electrode (2) was attached to a fixed test rig plate (4), the upper electrode was attached to the Velostat^®^ using a thin adhesive film. In the Velostat^®^ films comparison experiment, the used film was very thin and followed the shape of the sensing surface, but did not change its thickness, so its influence on the sensor characteristics could be neglected. Furthermore, it is worth to mention that under real conditions, a Velostat^®^-based sensor should be covered with a coating similar to the film we used to protect the material from external influences and minimally affect the material characteristics.

Electrodes (2 and 3) were located perpendicularly to each other; the contact area between the electrodes was 25 mm^2^. The force was applied to a movable plate (5) in the normal direction. The Velostat^®^ material (1) between the contacts (2 and 3) was also affected by the same compressive force value. Resistance was evaluated by measuring the voltage between electrodes (2 and 3) and calculated from the electric circuit parameters (Equations (1) and (2)). Signals were converted to digital ones by the analogue–digital converter (ADC) and transmitted through an interface circuit to a data acquisition unit (DAQ) (both are parts of NI USB-4432 device from National Instruments, Austin, TX, USA). The data were stored and processed by a personal computer.

### 2.2. Methodology

#### Evaluation of Mechanical Characteristics of the Developed Sensor

**Figure 2:**
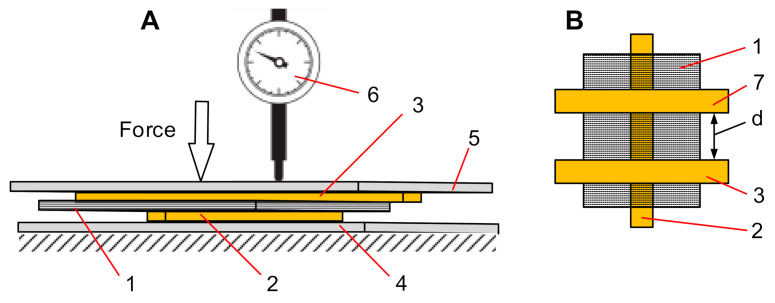
(**A**) Test rig system for the displacement dependence on compressive force measurements, where: 1—pressure-sensitive conductive composite material—Velostat^®^, 2—bottom electrode, 3—permanent top electrode, 4—test rig frame, 5—top plate, 6—indicator. (**B**) Test structure, which was used for the displacement dependence on compressive force measurements, based on perpendicularly placed electrodes deposited on opposite site of Velostat^®^, where: 7—movable top electrode, d—distance.

The fresh sheet of the Velostat^®^ was placed in the test rig (Figure 1A) and compressed by the force in normal direction between two electrodes at their contact point (Figure 2A). Deformation of the material was measured as linear displacement using a dial indicator (measurement ranges from 0 to 2 mm with a resolution of 0.005 mm) supported on the movable test rig plane (Figure 2A). The movable test rig plane in a normal direction was loaded by the force gradually varying from 0 to 2.94 N with a step-up increment of 0.42 N every 5 s. The force was gradually increased until it reached the maximum value and then reduced by the same sequence. Loading cycles were repeated after 60 s. Displacement values were measured during each step of the load.

Applying described loading in a cyclic manner, we performed several experiments under different conditions. The first set of measurements was performed using ‘fresh’ (mechanically untreated) material. The experiment was repeated 7 times. After this test the Velostat^®^ material was left ‘to relax’, meaning it was not subjected to a force impact (the force was reduced to 0 N). The second set of measurements was performed with the same (already used in tests) material after 60 min by the same methodology. The third experiment was performed after keeping the Velostat^®^ for 60 min loaded by a force of 2.94 N. Then, the force was reduced to 0 N, and the experiment was repeated using the same methodology.

**Figure 3:**
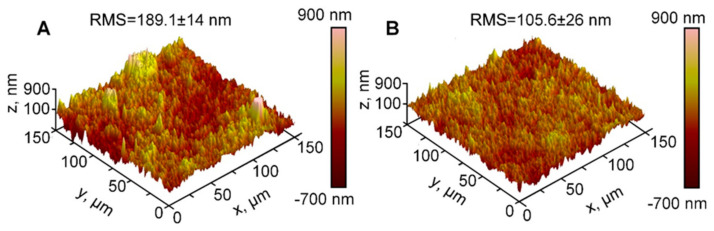
AFM images of surface roughness of: (**A**) fresh Velostat^®^ and (**B**) used Velostat^®^. RMS—roughness parameter root mean squared, calculated using the method described elsewhere [35].

Moreover, for a better understanding of the material behavior under loading conditions and to define factors causing plastic deformation, fresh and used material were investigated using Atomic Force Microscope (AFM). The topography of the Velostat^®^ (Figure 3) was measured with Veeco Bioscope II Atomic Force Microscope equipped with silicon nitride probes of NP series (Veeco MLTC) with the triangular cantilever of the spring constant below 0.1 N/m with the four-sided pyramidal tip from 20 to 60 nm radius.

### 2.3. Evaluation of Sensor Electric Properties

To evaluate the sensor’s electric properties, we have performed a set of experiments which allowed us to define the main characteristics such as sensitivity, response time and transversal resolution.

#### 2.3.1. Evaluation of Sensor Sensitivity

Sensor sensitivity, which defines the relation between sensor resistance and the applied load was determined when the sensor was pressed with a certain force and then was released. Such an experiment allowed us to define sensor hysteresis as well. Two types of Velostat^®^ (fresh and used) were used to conduct research and compare the obtained results. The used Velostat^®^ refers to a material that has already been used. Meanwhile, the fresh Velostat^®^ refers to a material that has not been used in experiments before.

Experiments were performed using a previously described test rig (Figure 1). During the test, piezoresistive material was pressed with a force of 0.14 to 3.08 N with an increment of 0.42 N. Before the experiment, when the material of the test sensor was already loaded with 0.14 N, a period of 10 min was provided for the material to settle. The force was further increased every 8 min until a maximum force of 3.08 N was reached. Such long time period was selected to have a possibility for a better sensitivity drift observation. After reaching the maximum force value, the force was reduced to 0.14 N in the same manner. The experiment was repeated 11 times.

The sensor response was measured as a voltage between contacts using a measurement circuit (Figure 1C) and Ni 4431 (National instrument, Austin, TX, USA) data acquisition system. Measured data in the form of the voltage were recorded using the SignalExpress software package and processed by Origin program. The measured voltage was converted into Velostat^®^ layer resistance using Equation (2).

#### 2.3.2. Evaluation of Sensor Response Time

Sensor response time defines the amount of time required for the sensor signal to stabilize after the load changes. This characteristic defines sensor response time and shows its potential for measurement of dynamic processes. The response time of the Velostat^®^ to the typical step load of 15 N (size of the contact area was 48 mm^2^) was defined in [34]. However, due to the nonlinear Velostat^®^ properties, the conventional method does not fully evaluate the behavior of the material. We performed an extensive investigation of the Velostat^®^ response time evaluating this characteristic under dynamic conditions, when instead of step loading, gradually increasing load was applied.

Sensor response time was defined from raw data obtained by evaluating sensor sensitivity. Response time after each increase of the load was defined using the ‘Rise time’ function, which is available in the Origin software.

#### 2.3.3. Evaluation of Transverse Sensor Resolution

At this stage, we investigated how the sensor voltage changes when one pair of the electrodes, which are deposited on the Velostat^®^, is shifted aside from the main pair of electrodes (Figure 2); this will allow us to evaluate the influence of force applied in the surrounding area to the measuring electrodes. This research will provide a background to the possible highest resolution of the tactile sensor with the Velostat^®^ material layer. In this case, only fresh Velostat^®^ material was used for the tests. Resistance measurements were performed with a Velostat^®^ layer fixed to the lower plate (fastening as in the previous test) with no force (0 N) and different compressive force values: 0.98, 2.24 and 3.08 N applied with random order. The distances between the contacts were changed in unloaded mode. The distances between the permanent and movable contacts were set to values of 1.5, 3, 6, 9, 12, 15 and 18 mm and were applied in random order. All experiments were repeated 11 times. Resistance average values were calculated for each of the 11 test results obtained with a differently loaded Velostat^®^.

### 2.4. Calculations

The most suitable way to obtain data from resistive sensors is to implement a voltage divider circuit consisting of the variable, which is the sensor itself, and additional reference resistors (Figure 1C). Using this circuit, the resistance of the sensor is obtained according to the following equation:

(1) $$R_{2}=\;\frac{V_{out}\;\cdot \;R_{1}}{V_{in}-\;V_{out}}\;$$

where *R*_1_ is the resistance of the reference resistor, *V_out_* is the output voltage from the measuring circuit, *R*_2_ is the resistance of the Velostat^®^ at the measurement point, and *V_in_* is the measurement input voltage applied to the circuit.

In our research, the input voltage *V_in_* was equal to 5.05 V and resistance of reference resistors *R*_1_ was equal to 11,000 Ω. The measured output voltage *V_out_* was transformed into material contact resistance *R*_2_ using the simplified formula:

(2) $$R_{2}=11000\times \left(\frac{5.05}{V_{out}}-1\right)\times 0.001$$

## 3. Results of Experimental Research

Fresh and used Velostat^®^ surface evaluation was performed by AFM (Figure 3). The AFM image reveals a way of material change after some load cycles. The changes in the surface topology demonstrated sufficient support area development due to lower roughness of used Velostat^®^. In our case, seven full load cycles caused a decrease of surface roughness from 189 to 106 nm. The decrease of surface roughness evaluated by RMS (root mean square) parameter [35] in 44% could be explained by the fracture of carbon particles located on the upper and bottom material surfaces and squeezing them deeper into the material by mechanical pressure.

After analyzing surface structure, mechanical material characteristics based on static deformation under load were evaluated. The evaluation procedure was performed by defining a dependency between the applied load and the deformation of the sensor. Deformation of the Velostat® was measured as described in the experimental methodology in Section 2.2.

**Figure 4:**
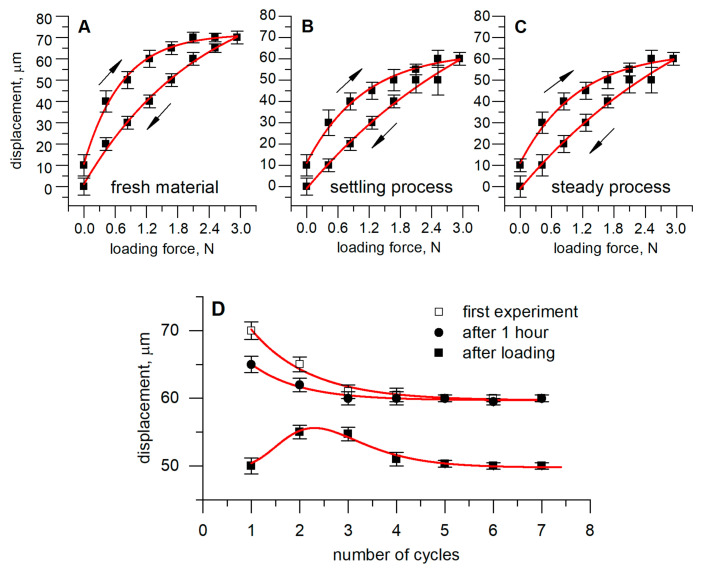
Dependence of sensor deformation on the applied load at different experimental conditions: (**A**) 1st cycle, new material; (**B**) 4th cycle, settling process; (**C**) 7th cycle, steady process; (**D**) displacement dependence on the number of experiments, performed at the beginning of the experiment, after 1-h lasting break during which sensor was unloaded, and after 1-h break during which sensor was loaded by 2.88 N.

The first set of experiments (Figure 4A–C) was performed using a fresh piece of Velostat^®^ material, which was cyclically loaded/unloaded. The highest compressive deformation was defined by analyzing the vertical displacement of the movable test-rig plate 5 (Figure 1B), measured during the first cycle (Figure 4A). The material showed nonlinear contact stiffness as a response to pressure. The initial test-rig movable plate displacement reached 70 µm under the load of approximately 2 N. Continuing the cycling load, displacement gradually decreased, and after the 5th cycle, it reached stable characteristics (Figure 4B). Displacement under a load of 2.4 N was equal to 60 µm, and in the next cycle, this value remained stable (Figure 4C). Identical measurements were repeated after keeping the sensor loaded at 2.88 N for 1 h. Averaged results representing deformation under the maximum tested load (by 2.88 N) are presented in Figure 4D. As can be seen, the resulting displacement drifted from the initial loading. This phenomenon could be explained by analyzing the effect of plastic deformation—after keeping sensor loaded for 1 h at a 2.88 N load, its material was compressed by 50 µm. After removing the load, the material recovered, and the second and third loading cycle showed deformation equal to 55 µm, which is similar to values (60 µm) obtained with the material used under the cycling load. During further cycling, the deformation value decreased 50 µm as it was during the first cycle after unloading. Such results show that the material did not recover to its original properties after long-term loading, or a time period of 60 s between unloading and the start of the experiment was too short. This proves that when implementing Velostat^®^ in professional sensors, it is necessary to take into account the type of load, perform additional tests and develop a compensation algorithm for the cases where the sensor is exposed to a long-term maximum load.

**Figure 5:**
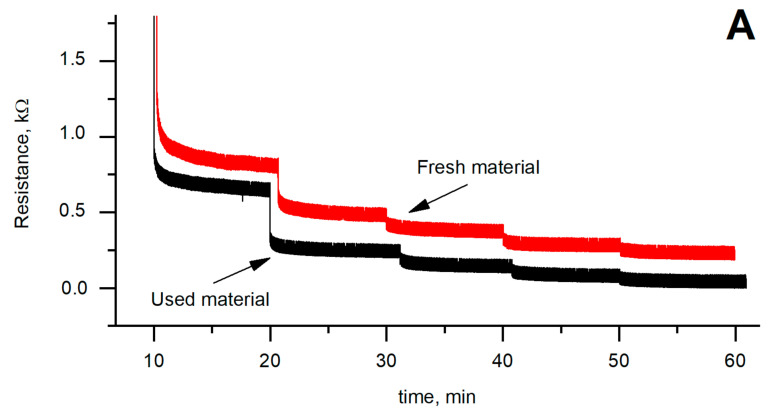

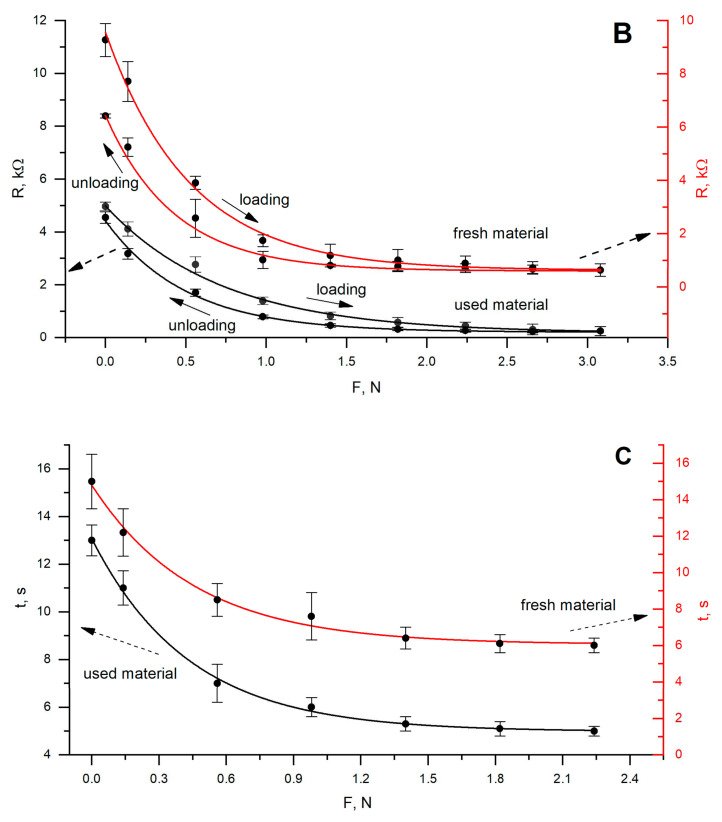
(**A**) Resistance dependence on time. Material was pressed with a force of 0.14 to 3.08 N with an increment of 0.42 N. The force was further increased every 8 min. (**B**) Dependencies between the load and the resistance of a used and fresh material. (**C**) The response time of used and fresh Velostat^®^-based materials.

The results of sensor sensitivity evaluation (Figure 5A,B) show the nonlinear behavior of the sensors, which corresponds to its mechanical properties. It is seen that in a comparison between fresh and used material, the resistance of fresh material is higher. It can be stated that this change is caused by the previously described effects: applied load causes a decrease of the material thickness, influence the surface roughness and the squeeze the carbon grains deeper into the material. This affects the decrease of the distance between the conductive particles and increases the conductivity of the material.

The experiment of stabilization of the used and fresh Velostat^®^-based materials shows: when the higher force is applied to the Velostat^®^, then the settling time of the sensor is shorter (Figure 5C). Such behavior is mainly defined by material mechanical properties and could be explained by hyperelastic stress–strain behavior, corresponding to the Neo–Hookean material or Mooney–Rivlin material and the influence of internal friction. Internal frictions try to resist the applied force. If the applied external force is significantly higher, the deformation process is faster and, correspondingly, stabilization time becomes shorter. A comparison among the Neo–Hookean model, Mooney–Rivlin model, and Ogden model for chloroprene rubber is presented in [36].

The results in Figure 3, Figure 4 and Figure 5 show correlations between Velostat^®^ mechanical and electrical properties. A decrease of the surface roughness detected using AFM (Figure 3B) explains the sensor mechanical behavior and the shift of dependencies in Figure 4, especially deformation after loading the sensor for one hour. The obtained mechanical characteristics correspond to the sensor resistivity. The lower surface roughness of used material leads to lower resistivity as is seen in Figure 5A. The drift observed in the electrical and mechanical characteristics allows us to conclude that after 5–7 load cycles, changes in the material structures slow down significantly and characteristics become more stable.

**Figure 6:**
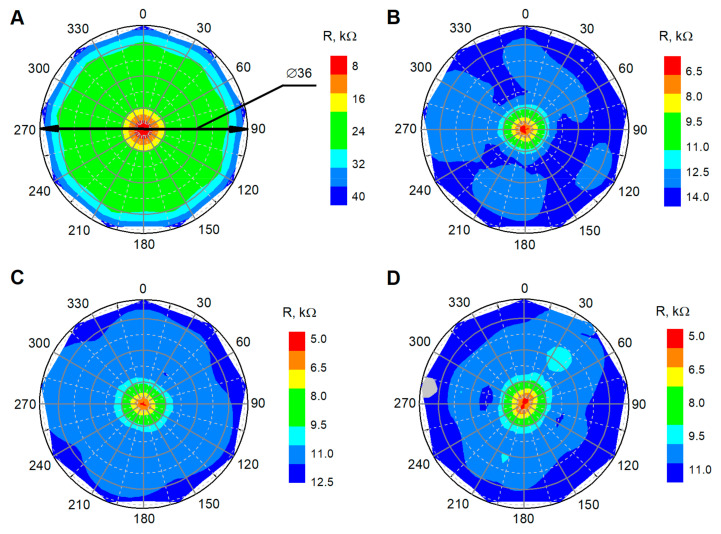
Resistance distribution of (**A**) 0.14 N, (**B**) 0.96 N, (**C**) 2.20 N and (**D**) 3.02 N load, 2D view.

Mechanical aspects affecting electrical properties are one of the most important elements influencing the parameters to control the physical, mechanical and processing properties of plastic/polymeric materials. In such a situation, it is important to know how loads applied aside from the electrode area influence Velostat^®^ resistance indications. The distribution of the side load induced resistance fluctuation is depicted in Figure 6. This test reveals the dependency of a distance and applied the force value to the distortion of the resistance indications.

The electrical properties of the sensitive composite material highly depend on its polymer-based structure deformation under a permanent load. The relaxation time and degree after load removal is a key aspect for implementing Velostat^®^ as a sensing-material in any type of sensors. Moreover, the stability of the results in steady-state and cyclic applications of load define the level of reliability for such sensors. Existing hysteresis loops in the load cycle and the settling of resistance parameters after some cycles indicate the high value of internal friction; therefore, the use of the sensor in dynamic applications requires additional research.

For a force or pressure sensors matrix, the load at the electrode contact area affects the surrounding area and becomes crucial in the development of such types of sensors. Therefore, sparse resolution of the sensor was also evaluated by experimental research.

The provided experimental results point to the fact that even low values of applied force aside measuring contacts induce change of resistance between them. Influence increases with the increase of force and decreases as the distance increase. This situation opens the implementation field for tactile sensors, but our reported results indicate some important restrictions of such a force sensor matrix. The influence of an undesired current drift through the side has been observed, but with a higher distance from the loaded area, the electrical resistance increases due to the longer charge transfer path charge carriers meeting a higher number of obstacles through their paths.

## 4. Conclusions

The use of polyethylene-carbon composite (Velostat^®^) as a sensitive material for pressure, force and tactile sensors has many advantages: (i) small dimensions, because the thickness of the film is only 0.2 mm; (ii) possibility to produce a sensitive element of a required shape using simple production technology; (iii) possibility to produce arrays of the pressure/force sensors; (iv) Velostat^®^ is a flexible polymer-based material, which allows to create sensor of the desired shape. Such types of sensors with Velostat^®^ as a sensitive material also have features limiting the application field of these sensors. First, experimental research has shown that initial loading/unloading/reloading-based cyclic treatment of a sensor is required before starting the calibration of the sensor. The settling of Velostat’s^®^ properties is a clear process, but it will slow down the production process. Second, the characteristics of the material are depicted by a significant hysteresis process, which indicates high internal friction. High internal friction causes limitation in measurement of dynamically applied loads. However, the evaluation of dynamic properties of this composite material still requires additional research in the field of experimental mechanics, while implementing the material in time-dependent load sensing requires the evaluation of its generalized internal friction level. This generalized friction will cover few material effects, but for signal processing, it is much more efficient to reduce these effects on the value of friction.

Nonlinearity is typical for this type of piezoresistive sensor, but an indication of the measurement process will be compensated using data processing. Influence of proximity load creates a controversial condition, because higher loads have more significant influence to the contact area between electrodes and the polyethylene-carbon composite. The main challenges during our research were based on the evaluation of the nonlinear behavior of such material; the matrix resolution is cross-linked to the applied load, the compression and relaxation of the material is also related to the value of the load, and the resistance shift from the new material application to the settling point also depends on the load and its duration.

The performed research opened possibilities to create dense sensor matrixes. The obtained resolution for load measuring can reach 3 mm in the distance between electrodes, and tactile sensor density definition will come in the next report. The multiple sensor loading case showed the possibility to settle Velostat^®^ materials and of using such sensors as lifelong and reliable devices. The researched mechanical properties of the material and the load–unload deflection behavior revealed sensor reliability and signal drift issues. Finally, the results of our research can provide data for sensor output correction using a model-based approach. Nevertheless, the sensor matrix can be created using this polyethylene-carbon composite, but the resolution of the developed sensor will depend on the measured load value. The tactile sensor will have the highest resolution, while force sensors cause limits of resolution that depend on the loading force. The performed research opens a broad perspective of implementing Velostat^®^ in force sensors, because they are inexpensive, flexible, impact safe, and suitable to be used for the development of wearable sensors, but their dynamic properties still require additional specialized research, which is planned in the future.

## Figures and Tables

**Table 1:** Piezoresistive sensors design and their characteristics.

Material, Electrode	Measured Quantity, Contact Area, mm^2^	Evaluated Characteristics	Ref.
Velostat^®^, copper	Force (0–3 N), 25	Sensitivity, hysteresis, mechanical compression, response time, transverse resolution	This work
Velostat^®^, -	Pressure (0–250 kPa), 100	Sensitivity, repeatability, time drift, hysteresis, dynamic response, sensitivity threshold	[37]
Velostat^®^, copper	Pressure (0–0.27 kPa), 450	settling time, dynamic response	[10]
Velostat^®^, silver-covered polyamide	Pressure (0–1000 kPa), 100	Sensitivity,	[38]
Polyurethane film, Aluminum foil	Pressure (0–650 KPa), 4900	Sensitivity, cycle test	[39]
Graphene nanoplatelets and carbon nanotubes, PMMA and PVDF, Silver	Pressure (0–100 000 kPa), -	Sensitivity,	[40]
Linqstat, copper	Force (0–5 N), -	Resolution	[41]
Velostat^®^, aluminum foil	Force (0–40 N), 10150 and 2040	Replicability, repeatability	[9]
Velostat^®^, copper	Force (10–100 N), 219.8	Sensitivity,	[42]
LDPE, Shieldex NoraDell woven fabric sheets	Pressure (1–10 kPa), 342	Sensitivity, recovery time	[43]
Velostat^®^, several different materials	Force (0–20 N), 1000	Sensitivity,	[44]
Velostat^®^, Satatex Techniktex P-130	Force (2–136 N, 2–173 N, and 2–210 N), 56.25	Cyclic, time	[45]
Velostat^®^ and EX-STATIC fabric, copper	Force (0–500 N), 100	Sensitivity, time drift	[46]
Polyethylene Terapthalate, silver	Force (0–53 N), -	Sensitivity, time drift	[47]
Velostat^®^, -	Force (0–16 N) -	Sensitivity, time drift	[34]
